# Soil nematode community and crop productivity in response to 5-year biochar and manure addition to yellow cinnamon soil

**DOI:** 10.1186/s12898-020-00304-8

**Published:** 2020-07-16

**Authors:** Xiaodan Liu, Dengxiao Zhang, Huixin Li, Xiuxiu Qi, Ya Gao, Yibo Zhang, Yanlai Han, Ying Jiang, Hui Li

**Affiliations:** 1 College of Resources and Environment, Henan Agricultural University, Zhengzhou, 450002 China; 2 College of Resources and Environmental Sciences, Nanjing Agricultural University, Nanjing, 210095 China

**Keywords:** Biochar, Manure, Nematode community, Yellow cinnamon soil, Yield

## Abstract

**Background:**

Manure and biochar soil amendments have shown many benefits to soil quality and crop productivity. This study aimed to reveal the effects of biochar and manure applications on soil fertility improvement and crop productivity in yellow cinnamon soil.

**Results:**

This study based on a 5-year field experiment. Four treatments were designed, included the control (CK), biochar amendment, manure amendment, and both biochar and manure amendment (BM). The results showed that: after five years, both biochar and manure treatment improved soil structure by increasing soil mean weight diameter (MWD), and soil water and nutrient supply was also increased by increasing the contents of water content, available potassium and available phosphorus. The productivity was also enhanced as wheat yield under the biochar, manure, and BM treatments increased by 3.59–11.32% compared with CK. In addition, biochar and manure treatment increased soil microbial biomass carbon (MBC) by > 15%, and soil total nematode abundance was significantly increased. Furthermore, the nematode community structure was significantly affected by biochar and manure treatment, dominant trophic group in CK was herbivores, but bacterivores were dominant in the biochar and manure treatments. The distribution of nematode genera was closely related to soil chemical properties and microbial biomass. Increases in the Shannon's diversity index, and decreases in the dominance index and summed maturity index after the 5-year treatment indicated a sustainable soil ecosystem after the biochar and manure applications.

**Conclusions:**

These findings indicate that biochar and manure result in better soil quality and increased productivity in yellow cinnamon soil.

## Background

Organic amendments, such as manure and straw, can provide many benefits in agricultural production, including soil fertility improvement and crop yield enhancing across different farming systems [1]. Additionally, biochar, mostly from the pyrolysis of agricultural biowastes, has been shown to provide a soil conditioner benefit to soil quality and productivity [2]. It has been suggested that both manure and biochar soil amendments affect soil nutrient retention [3, 4], soil structure formation [5, 6], and microbial dynamics [7, 8] in agricultural systems. The ability of biochar to enhance soil fertility has been demonstrated in many types of soils. Additionally, biochar provides habitat in which microbes and soil animals can escape predators and obtain substrates and mineral nutrients [9], which has shown a positive effect on soil organisms such as microbial communities. Zhang et al. [10] collected current published literature and reviewed the responses of soil microorganism community structure and activities after biochar amendment, they found that soil microbial biomass and activities were increases with biochar amendment, and microbial community structure was improved in the long-term condition. Studies of the effects of biochar addition on soil fauna are much less abundant than those on microorganisms. Van Zwieten et al. [11] conducted a behavioral experiment and found that earthworms preferred biochar-amended soil over non-biochar amended soil in a Ferrosol. While Weyers and Spokas [12] determined that biochar amendment had a negative impact on earthworm population density and total biomass after short-term biochar application. In addition, nematode is the most abundance animal in soil, and Zhang et al. [13] found that soil nematode trophic groups were sensitive to biochar addition. The variations of diversity and functional indices of soil nematode provide insights into soil fertility and ecosystem functioning [14, 15]. While most studies on soil nematodes have focused on plant-parasitic nematodes, especially the nematode species damage crops. Nevertheless, knowledge on the responses of soil nematode community structure and ecology indices to biochar addition remains a gap.

The application of manure directly adds nutrients to soil and improves soil structure, thereby increasing nutrient retention and water holding capacity. These changes can stimulate microbial growth and activity, which facilitate soil nutrient cycling and benefit crop production [16]. The effect of organic fertilizer soil amendment on nematode community structure has been widely studied [17–19]. Generally, application of organic fertilizer increases soil total nematode abundance and species richness. Liu et al. [17] conducted a meta-analysis and indicated that organic fertilizer treatment increased total nematode abundance by 37%, whereas mineral fertilizer had no effect, compared to untreated soil. Liang et al. [20] found an approximately 50% higher species richness with manure treatment than with nitrogen fertilizer treatment based on a 20 years’ field experiment. The effect of organic amendment on soil nematode community was varies with the treatment years. As short-term organic treatment mainly impacts on the function of soil nematodes [21], and long-term application can increase total nematode abundance and diversity [19, 22]. In addition, the change of nematode species was difference according to organic amendment. Villenave et al. [23] indicated that pig manure amendment increased plant-feeding nematodes, whereas crop straw amendment increased the abundance of fungus-feeding nematodes. And Thoden et al. [24] noted that manure and compost application to the soils increased the populations of plant-parasitic nematodes, while Takahiro et al. [25] indicated that organic amendment could suppress soil plant parasitic nematodes. This may be related to organic fertilizer types or soil conditions [26]. From soil nematode ecological indices, manure amendment has been shown to benefit the nematode community and create a well-structured and complex soil nematode community, as organic materials applied to soils tend to increase total nematode abundance, diversity and enrichment index, but decrease the summed maturity index (∑MI) and structural index [17].

Nematodes are the most abundant type of animal on Earth [27]. They occur in almost all ecosystems and occupy a range of trophic groups, including bacterivores, herbivores, omnivore predators, and fungivores [28]. Nematodes play important roles in the soil nutrient cycle, plant growth and health, and soil food web stability [29]. The ecological indices of soil nematodes are sensitive to agricultural practices such as fertilization. Li et al. [30] found that manure treatment disturbed soil food webs via nematode faunal analysis. So the activity of soil nematode would alter the soil microbial community, nutrient biogeochemical process, and finally effect on crop growth [31]. Therefore, studies on the response of nematode community structure to agricultural management are helpful for assessing its effect on soil quality and soil productivity.

Yellow cinnamon soil is a widespread soil type in China. However, its poor soil structure and lower fertility have become restrictive factors limiting crop productivity [32]. Recently, large inputs of mineral fertilizer have intensified the problems. This study was based on a 5-year field experiment conducted in yellow cinnamon soil, biochar and manure treatment was conducted in the experiment, and the readily available waste of chicken manure and peanut was selected as the feedstock of organic fertilizer and biochar, respectively. Soil nematodes were chosen as the indicator organism, and we aimed to reveal the effects of biochar and manure applications on soil productivity and soil quality. We hypothesized that the application of biochar and/or manure application would increase soil fertility by improving the soil structure and increase the nutrient supply of yellow cinnamon soil, which provide a better living micro-environment for soil ecosystem and would improve the community structure of soil nematodes and ultimately enhance soil productivity.

## Results

### Soil physicochemical properties

Two-way ANOVA showed that soil available N, available P, SOC, and mean weight diameter (MWD) were significantly affected by both the biochar and manure amendment, compared to CK (*P* < 0.05) (Table 1). Biochar amendment increased the SOC and MWD by 15.72% and 5.82%; manure treatment increased them by 13.32% and 14.32%, respectively; and BM treatment increased SOC and MWD from 12.59 g kg^−1^ and 0.40 mm to 15.33 g kg^−1^ and 0.46 mm, respectively. Compared with CK, biochar treatment increased the available P by 17.73% and reduced the available N by 7.67%. Manure significantly increased the available P (*P* < 0.01), although there was no significant difference in available N.

**Table 1:** Effect of different fertilization treatments on soil physicochemical properties

Treatment	Soil water content (%)	pH (H_2_O)	MWD (mm)	Available N (mg kg^−1^)	Available P (mg kg^−1^)	Exchangeable K (mg kg^−1^)	SOC (g kg^−1^)
CK	11.33 ± 1.78b	5.60 ± 0.35a	0.40 ± 0.00c	120.03 ± 11.44a	22.85 ± 8.07c	167.18 ± 3.94a	12.59 ± 0.29c
Biochar	13.13 ± 0.30a	5.59 ± 0.48a	0.43 ± 0.01b	110.82 ± 2.91b	26.90 ± 3.78b	187.65 ± 33.38a	14.57 ± 0.39b
Manure	13.43 ± 1.33a	5.46 ± 0.32a	0.46 ± 0.00a	120.67 ± 6.34a	40.91 ± 6.53b	173.95 ± 24.13a	14.26 ± 0.20b
BM	13.29 ± 0.36a	5.62 ± 0.38a	0.46 ± 0.01a	106.91 ± 3.24b	62.61 ± 14.88a	190.45 ± 41.21a	15.33 ± 0.28a
Two-way ANOVA							
B	ns	ns	ns	16.65**	5.80*	ns	79.57**
M	ns	ns	104.14**	ns	25.28**	ns	51.34**
B × M	ns	ns	11.57**	ns	ns	ns	7.08*

B, M and B × M means Two-way ANOVA result of Biochar, Manure and BM treatment, respectively. The Different lower-case letters represent significant differences among fertilization treatments, *P* < 0.05

The same is for Tables 3 and 4

* Test significant at the 5% level (*P* < 0.05)

** Test significant at the 1% level (*P* < 0.01); ns: test non-significant at the 5% level

**Fig. 1:**
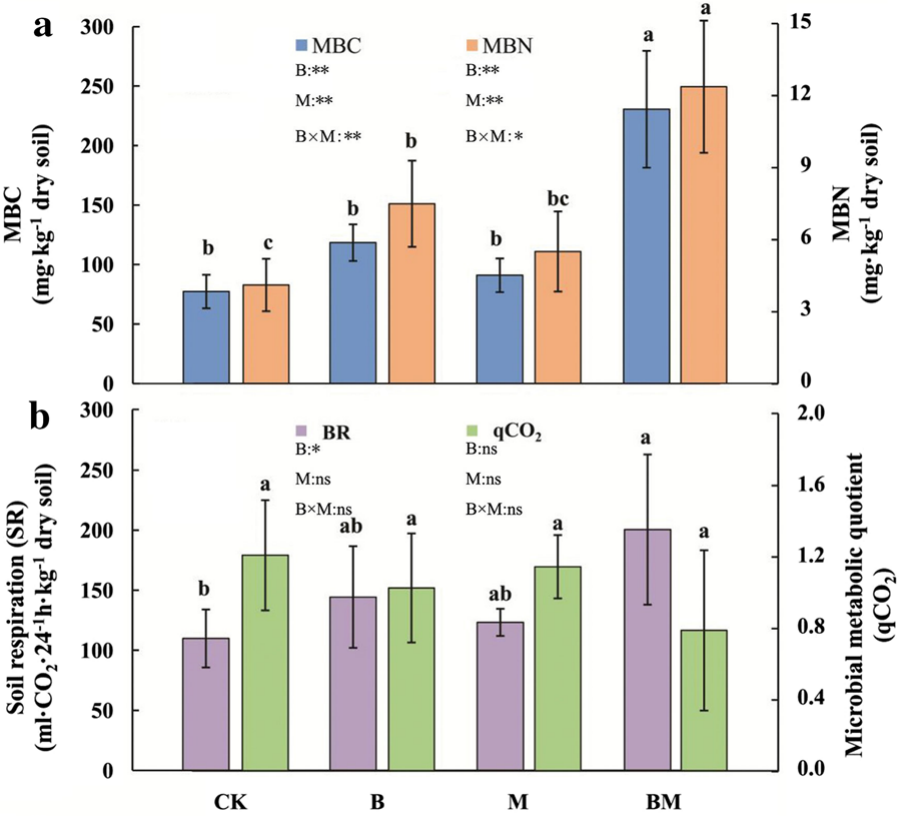
Effects of different treatments on **a** soil microbial biomass carbon (MBC) and microbial biomass nitrogen (MBN) and **b** soil respiration (SR) and microbial metabolic quotient (qCO_2_). The results of two-way analysis of variance on the effects of biochar (B), manure (M), and their interaction (B × M) on MBC, MBN, SR, and qCO_2_ are also shown in the figure. Error bars represent standard deviation. Different lowercase letters represent significant differences among fertilization treatments (*P* < 0.05). *P < 0.05; **P < 0.01; “ns”, not significant at the 5% level. The same notation applies below

The two-way ANOVA for microbial biomass carbon (MBC) and microbial biomass nitrogen (MBN) showed significant effects with biochar and manure and also showed a significant interaction (*P* < 0.05) (Fig. 1). The Biochar and Manure treatments increased MBC by 53.15% and 17.66% compared with CK, respectively, and BM treatment greatly increased MBC by 198.19%. Biochar strongly increased soil respiration, and showed an interaction with Manure treatment. The qCO_2_ ranged between 0.95 and 1.45, there was no significant difference between treatments.

### Soil nematode abundance

In this study, 25 nematode genera were identified. The abundances of the different trophic groups are shown in Table 2. The 25 nematode genera belonged to 16 families: 4 families and 10 genera of bacterivores, 5 families and 5 genera of fungivores, 4 families and 4 genera of herbivores, and 2 families and 6 genera of omnivore-predators. The dominant trophic group of CK was herbivores, with an abundance up to 44.22%, but the dominant trophic group of the Biochar, Manure, and BM treatments was bacterivores, with abundances of 49.22%, 54.90%, and 56.69%, respectively. The dominant genera of CK were *Eucephalobus* (12.58%), *Cephalobus* (10.21%), *Aphelenchus* (10.39%), *Pratylenchus* (28.74%), and *Tylenchorhynchus* (11.04%); those of the Biochar treatment were *Eucephalobus* (15.02%), *Pratylenchus* (13.98%), and *Tylenchorhynchus* (12.21%); those of the Manure treatment were *Eucephalobus* (10.58%), *Acrobeloides* (20.26%), and *Tylenchorhynchus* (13.29%); and that of the BM treatment was *Acrobeloides* (13.33%). In addition, the applications of biochar and manure increased the relative abundance of bacterivores and reduced the relative abundance of herbivores (Table 2). In particular, Biochar, Manure, and BM increased the relative abundance of bacterivores from 34.04% in CK to 49.22%, 54.90%, and 56.69%, respectively. Additionally, they reduced the relative abundance of herbivores by 16.75%, 17.56%, and 27.74% compared to CK, respectively.

**Table 2:** Effect of different fertilization treatments on the proportion of soil individual nematode taxa (Mean abundance, percentage)

Trophic groups	Family	Genus	*Abbr.*	*c*-*p* value	Fertilization treatments			
					CK	Biochar	Manure	BM
Bacterivares	*Cephalobidae*	*Chiloplacus*	*Chil.*	2	1.55	0.00	0.00	0.00
		*Eucephalobus*	*Euce.*	2	12.58*	15.02*	10.58*	3.93
		*Acrobeles*	*Acro.*	2	2.53	9.43	6.34	3.65
		*Acrobeloides*	*Acrob.*	2	5.57	4.14	20.26*	13.33*
		*Cephalobus*	*Ceph.*	2	10.21*	3.95	2.89	8.45
	*Rhabditidae*	*Protorhabditis*	*Prot.*	1	0.67	3.28	1.95	7.37
		*Mesorhabditis*	*Meso.*	1	0.61	6.20	5.36	7.40
	*Panagrolaimidae*	*Panagrolaimus*	*Pana.*	1	0.00	5.88	5.76	5.80
	*Alaimidae*	*Alaimus*	*Alai.*	4	0.32	0.67	0.31	1.70
	*Plectidae*	*Chronogaster*	*Chro.*	2	0.00	0.66	1.43	5.07
Fungivores	*Tylenchidae*	*Ditylenchus*	*Dity.*	2	2.18	1.61	2.78	3.24
	*Aphelenchoididae*	*Aphelenchoides*	*Aphe.*	2	3.75	3.25	2.23	5.62
	*Aphelenchidae*	*Aphelenchus*	*Aphel.*	2	10.39*	8.08	8.56	9.67
	*Seinurinae*	*Seinura*	*Sein.*	2	0.00	2.60	0.51	1.88
	*Tylencholaimidae*	*Tylencholaimus*	*Tyle.*	4	0.00	0.00	0.82	0.00
Hebivores	*Tylenchidae*	*Tylenchus*	*Tylen.*	2	0.00	0.65	0.00	0.00
	*Pratylenchidae*	*Pratylenchus*	*Prat.*	3	28.74*	13.98*	8.14	3.18
	*Hoplolaimidae*	*Helicotylenchus*	*Heli.*	3	4.44	0.63	5.23	5.94
	*Tylenchorhynchidae*	*Tylenchorhynchus*	*Tyl.*	3	11.04*	12.21*	13.29*	7.36
Omnivore-predators	*Dorylaimoididae*	*Dorylaimoides*	*Dory.*	4	0.00	0.33	0.31	0.73
	*Dorylaimidae*	*Thorneella*	*Thor.*	4	0.00	0.00	1.13	1.35
		*Aporcelaimus*	*Apor.*	5	1.90	6.76	0.91	3.60
		*Pungentus*	*Pung.*	4	0.63	0.33	0.00	0.73
		*Mesodorylaimus*	*Mesod.*	5	2.89	0.00	0.00	0.00
		*Discolaimus*	*Disc.*	4	0.00	0.33	1.19	0.00

* The dominant genara, account for over 10% of soil nematode community. Values are nematode genera relative abundance (%)

**Fig. 2:**
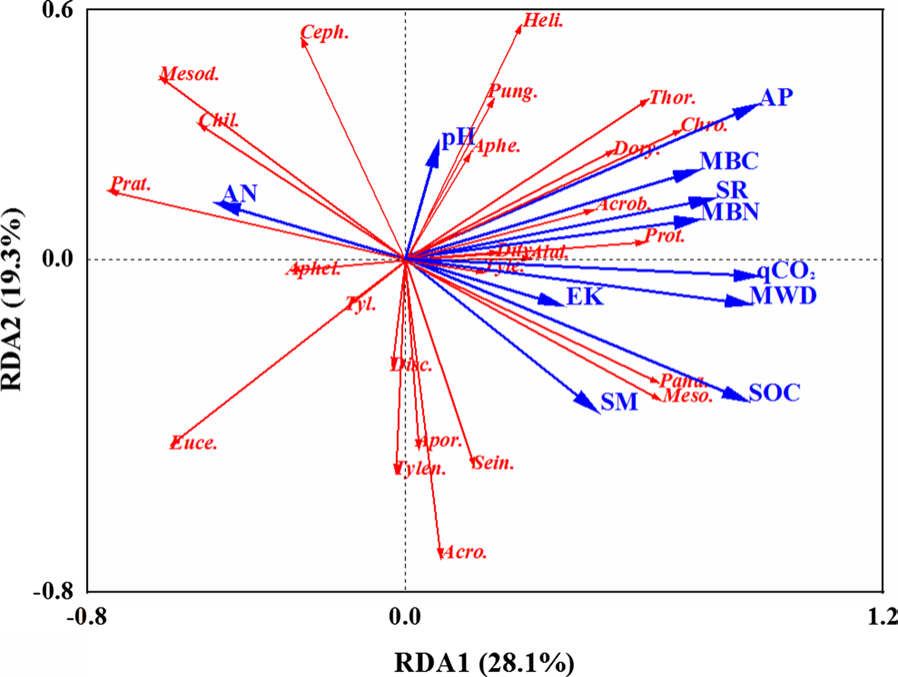
Redundancy analysis (RDA) diagram of the relationship between soil nematode assemblages and soil environmental factors

As illustrated in Fig. 2, the first two RDA axes showed high eigenvalues (7.02 and 4.82, respectively) in comparison to the values of subsequent axes, and explained 28.1% and 19.3% of the variation in species composition environment relationship, respectively. The composition of the nematode community was clearly discriminated among the different fertilization treatments, and the soil basic properties of available P, SOC, MWD, and soil microbial biomass and activity were closely related to the distribution of nematode genera. Furthermore, the RDA analysis exhibited an association between *Protorhabditis* and MBC, MBN, BR, and qCO_2_. Positive correlations were observed between *Chronogaster* and available P and between *Mesorhabditis* and SOC. And there was a negative correlation between *Pratylenchus* and MWD.

**Fig. 3:**
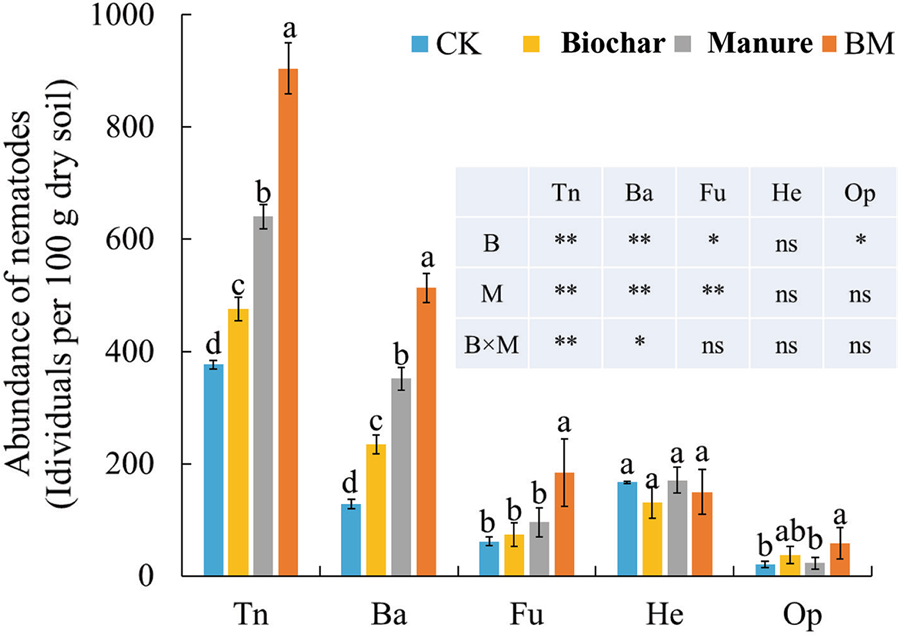
Effects of different fertilization treatments on nematode trophic groups and total nematodes. The results show a two-way analysis of variance of the effects of B, M, and their interaction (B × M) on nematode trophic groups and total nematodes

The biochar and manure treatments significantly affected the total nematode abundance (Tn) and the abundance of bacterivores (Ba) and also showed significant interaction (*P* < 0.05) (Fig. 3). In comparison with CK, the total nematode abundance in the Biochar, Manure, and BM treatments increased by 26.17%, 69.76%, and 139.88%, and the abundance of bacterivores increased by 82.48%, 173.80%, and 299.33%, respectively. Compared with CK, higher amounts of fungivores (Fu) were observed in Biochar, Manure, and BM. The application of biochar increased the number of omnivore-predators (Op), from 20 per 100 g dry soil to 37 per 100 g dry soil, but manure application showed no significant effect. Both the Biochar and Manure treatments showed no effects on herbivore (He) abundance.

**Fig. 4:**
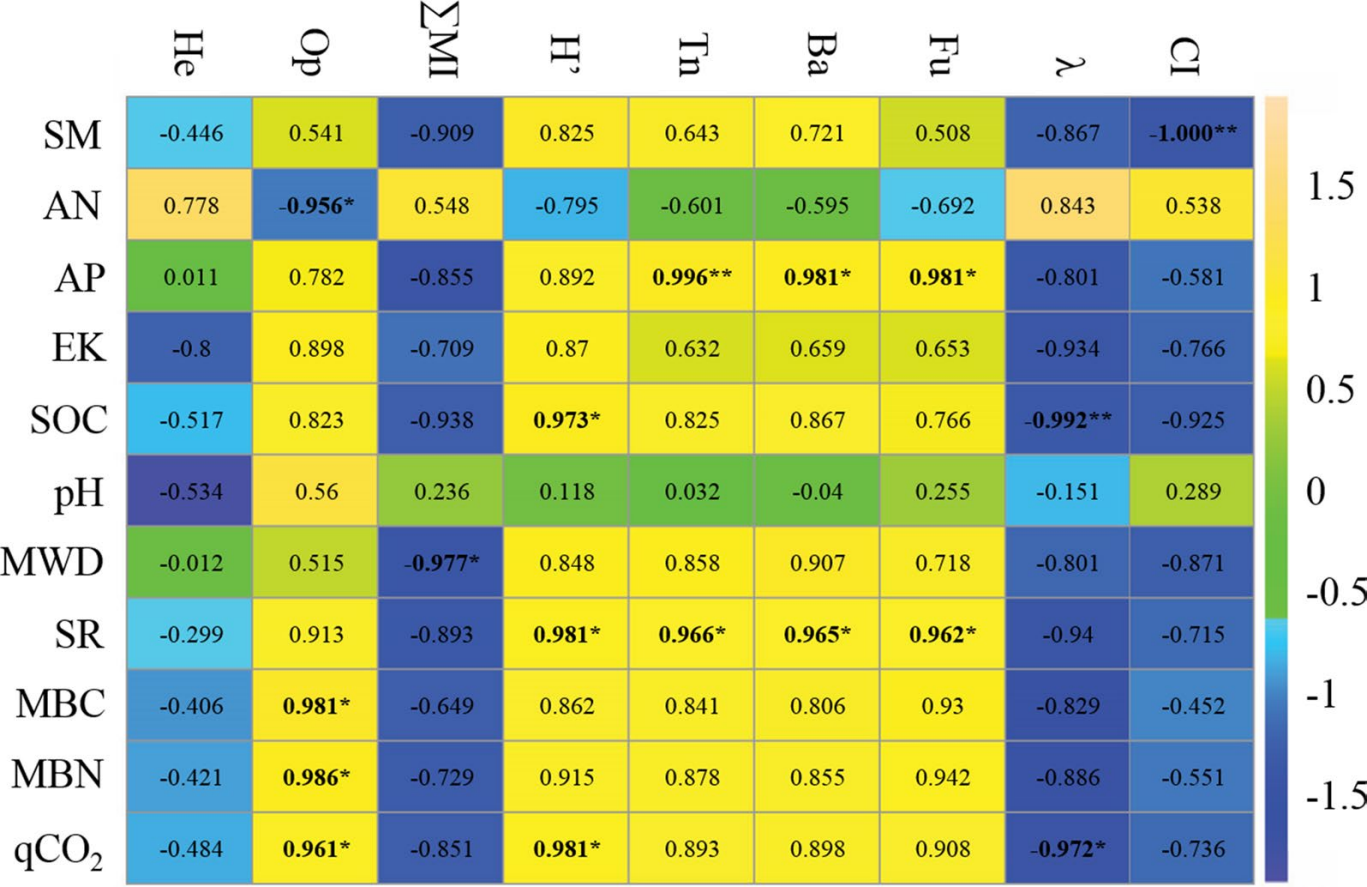
Pearson correlation coefficients between soil nematodes and soil factors

The correlation analysis showed that the total nematodes number and the bacterivore and fungivore abundances had a significant positive correlation with soil available P and SR (Fig. 4). There was no significant relationship between herbivore abundance and soil properties. The abundance of omnivore-predators showed a significant negative correlation with available N but a positive correlation with microbial carbon and nitrogen, and qCO_2_.

### Nematode community structure

The CI of CK was greater than 50, indicating that the decomposers were predominantly fungi (Table 3). However, the CI values of Biochar, Manure, and BM were less than 50, indicating that the decomposers were dominated by bacteria, and the correlation analysis showed that the change in CI was mainly related to soil moisture content (Fig. 4). The H’, λ, and ∑MI were significantly affected by the biochar and manure treatments, but there were no significant interactions. In particular, the biochar and manure treatments significantly increased H’ but significantly decreased λ and ∑MI. The changes in H’ and λ were associated with SOC content and soil respiration, and there was a negative correlation between ∑MI and MWD.

**Table 3:** Effect of different fertilization treatments on nematode ecological indexes

Ecological indices	Summed maturity index (∑MI)	Shannon’s diversity index (H’)	Dominance index (λ)	Channel index (CI)
CK	2.59 ± 0.05a	2.25 ± 0.02c	0.14 ± 0.00a	76.41 ± 9.89a
Biochar	2.35 ± 0.07b	2.48 ± 0.10b	0.10 ± 0.01b	20.71 ± 7.97b
Manure	2.24 ± 0.10bc	2.47 ± 0.10b	0.11 ± 0.01b	21.00 ± 2.54b
BM	2.16 ± 0.03c	2.69 ± 0.07a	0.08 ± 0.01c	20.27 ± 8.41b
Two-way ANOVA				
B	17.41**	24.09**	52.12**	40.02**
M	50.19**	22.23**	63.36**	39.23**
B × M	ns	ns	ns	37.99**

### Crop yield

Table 4 shows the effects of the different fertilization treatments on wheat yield and yield components. The biochar and manure amendments had no effect on panicle number or thousand seed weight, but they significantly affected wheat yield. In comparison with CK, the Biochar, Manure, and BM treatments increased wheat yield by 7.25%, 9.40%, and 12.76%, respectively. Manure treatment significantly increased the seed number, which increased by 16.89% compared with CK.

**Table 4:** Effect of different fertilization treatments on wheat yield and yield components

Treatment	Panicle number (10^4^ panicle ha^−1^)	Seed number (gain panicle^−1^)	Thousand seed weight (g)	Yield (kg ha^−1^)
CK	461.67 ± 31.93a	39.07 ± 3.88b	48.64 ± 1.05a	7422.84 ± 277.07b
Biochar	448.33 ± 8.33a	43.57 ± 1.97ab	47.98 ± 0.99a	7961.30 ± 287.39a
Manure	440.56 ± 30.84a	45.67 ± 1.79a	47.69 ± 3.70a	8120.93 ± 101.00a
BM	474.44 ± 26.37a	44.37 ± 2.06a	46.86 ± 0.39a	8370.00 ± 175.91a
Two-way ANOVA				
B	ns	ns	ns	9.28*
M	ns	6.22*	ns	18.33**
B × M	ns	ns	ns	ns

**Fig. 5:**
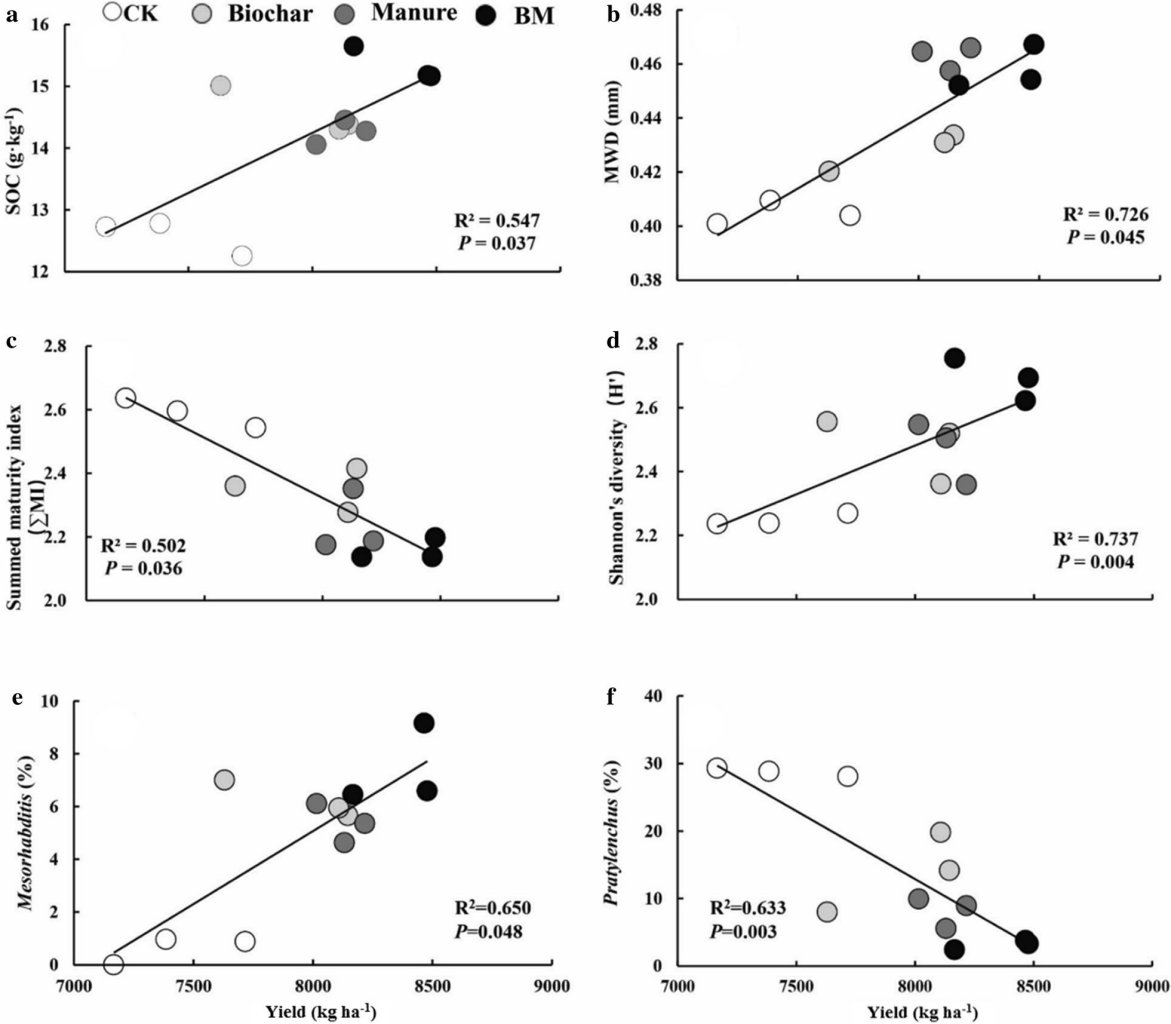
Relationships between soil factors and wheat yield. Note: R-squared (R^2^) and *P*-values were estimated from a linear regression model, and the best-fit line (——) is shown on the graph

Correlation analysis showed a significant positive correlation between wheat yield and SOC (*P* = 0.037) and MWD (*P* = 0.045) (Fig. 5). There was a significant negative correlation between yield and summed maturity index ∑MI (R^2^ = 0.502, *P *= 0.036), but there was a significant positive correlation between the Shannon's diversity index (H’) and yield. This indicates that the decrease in the maturity and the increase in diversity of nematodes may be factors influencing the increase in wheat yield. Furthermore, there was a significant positive correlation between wheat yield and *Mesorhabditis* abundance (R^2^ = 0.650, *P* = 0.048) and a significant negative correlation with *Pratylenchus* abundance (R^2^ = 0.6504, *P* = 0.048), indicating that *Mesorhabditis* and *Pratylenchus* may be the key nematode genera that correlated wheat yield.

## Discussion

### Effects of biochar and manure amendment on soil nematode community composition

The current study identified 25 nematode genera after wheat harvest, and the predominant trophic groups and genera differed among treatments. Changes in soil nematode composition were associated with the soil food supply and soil organic matter decomposition [33]. The current study showed that the predominant trophic group under CK treatment was herbivores, particularly *Pratylenchus* and *Tylenchorhynchus*, which occupied 39.78% of the total nematode abundance. This may due to the decrease of the physiological resistance of the crop after sole application of mineral fertilizer [34], and the weaker crop roots would have been easily infected by insects such as herbivores, and provide food supply to herbivores. Conversely, previous studies reported that biochar and manure application increases the resistance of crops [35, 36]. And the composition of organic fertilizer and the labial component of biochar provide carbon source for soil organisms. Therefore, the increase in soil bacteria abundance and the increased of crop resistance following biochar [10] amendment and organic fertilizer [37] amendment led to a transition in predominant trophic group from herbivores to bacterivores (Fig. 3). Additionally, manure is more labile for microorganism decomposition than biochar, thus the percentage of bacterivores was higher under manure than biochar amendment. This was further supported by Elzobair et al. [7] that the effect of manure was greater than biochar on soil bacteria abundance increasing.

In this study, soil total nematode abundance increased significantly under both biochar and manure treatment. This was in agreement with Liu et al. [17], who integrated 54 relevant studies around the world and found that organic amendment input increased soil nematode abundance by 37–50%, whereas mineral fertilizer amendment produced no increase. Additionally, the response of soil nematode abundance was related to the quality of the organic amendment [23]. Manure is relatively more labile than biochar, which resulted in more energy and carbon being provided to the nematode assemblage, and the manure also released more nutrients to the soil nematodes after decomposition. This explains the higher nematode abundance under manure than biochar amendment. In addition, the change in soil nematodes should be partly attributed to the change in soil microbial biomass. As the primary decomposers in soil, microorganisms first metabolize organic matter and then transfer energy and carbon to higher trophic groups, including nematodes. Therefore, soil MBC and MBN contents, as well as bacterivore and fungivore abundances, were all increased under biochar and manure amendments, compared to CK. The change in soil nematodes is related to soil properties, particularly soil pH and water content [38, 39], so soil available N and available P were shown significantly correlated with the abundance of trophic groups of Op, Ba, and Fu in this study (Fig. 4). Improvement of these soil physicochemical properties in the yellow cinnamon soil in this study created an optimal habitat for nematodes. Simultaneously, the improvement in soil physicochemical properties created an optimal environment for crop root growth. So some researchers have expressed concern that the increases in root biomass with biochar [40] and manure [41] may create a better environment for the growth of herbivores that may damage crop growth. However, in this study, the abundance of herbivores in yellow cinnamon soil was not significantly affected by biochar or manure treatment, which suggests that some soil environmental mechanisms or plant physiological mechanisms may exist. This topic requires further research.

The amendment of biochar and manure had many benefits on the improvement of soil nematode community, as indicated by ecological indices such as CI and H’. Our study demonstrated that the H’ was significantly higher under biochar and manure treatments than under single mineral fertilizer treatment. The increases in soil nematode H’ was significantly related to the better soil properties, such as adequate soil moisture and higher organic matter content, as the biochar and manure provided a sufficient food supply and a suitable soil environment for organisms [42, 43]. In the current study, the change of channel index (CI) reflected the dominant decomposers under the biochar and manure treatments were bacteria and the dominant decomposers under the CK treatment were fungi. This indicates that biochar and manure applications increased the resource availability to the soil food web [44]. In addition, the decreases in dominance index (λ) and summed maturity index (∑MI) reflected an increase in soil nematode community stability [45]. Therefore, the improvements in the soil nematode community with biochar and manure amendment indicated a more stable and sustainable soil ecosystem in yellow cinnamon soil. Furthermore, nematodes are important indicators for soil monitoring, so a healthier soil ecosystem and a better soil quality was established with biochar and manure application.

### Effects of biochar and manure amendments on soil productivity

Many studies have reported that biochar improves soil fertility by promoting soil aggregation [46], reducing soil nutrient loss (Nguyen et al. [58]), and increasing soil water retention capacity [5]. Biochar has a large specific surface area and rich functional groups on the surface, which can improve soil structure and regulate the release of soil nutrients. We found that biochar application promoted the formation of soil aggregation, and the MWD increased by 5.82% after, which would increase soil aeration and decrease soil bulk density [47]. Moreover, the current results showed that soil available P was significantly increased by 17.7% after wheat harvest, and soil water content was also significantly increased. Therefore, our study demonstrated that biochar amendment can improve the fertility of yellow cinnamon soil.

Organic fertilizer amendment of soil also provides various benefits for crop growth, such as the improvement of soil structure [26], and the increase of soil nutrient availability and moisture retention [48]. Our results further demonstrated that organic fertilizer application increased soil moisture content, and soil MWD was increased by 15%, indicating that organic fertilizer application was benefit to soil water retention and soil structure formation. The application of organic fertilizer can increase soil CEC, and the stock of soil organic matter is crucial for nutrient retention and nutrient availability [49]. Thus soil available P was increased significantly in the manure treatment in this study. Most changes of soil properties were associated with biological processes, such as microbial biomass and enzymes activities. Miller et al. [50] and Thangarajan et al. [49] studies the effect of organic amendment on soil properties and reported that organic materials application to the soil could increase extracellular enzyme activity and soil microbial biomass, which further influenced soil nutrient recycling such as carbon, phosphorus and potassium. In the current study, we found that both soil MBC and MBN were significantly increased by manure amendment (Fig. 1). This was consistent with the result reported by Allison and Martiny [51] that the microbial community structure and activity were sensitive to organic fertilizer application.

The improvement of soil properties through organic fertilizer application leads to higher soil productivity, Especially soil organic content and MWD were significantly correlated with wheat yield (Fig. 5). A meta-analysis conducted by Luo et al. [1] showed that organic fertilizer amendment increased crop yields by 27% compared with mineral fertilizer. Our study indicated that manure amendment increased wheat yield by 9.4% in yellow cinnamon soil after 5 years’ manure treatment. Organic fertilizer decomposition occurs after the fertilizer mixed with soil, and nutrient release becomes more synchronized with crop demands [52] and reduced the nutrient loss which increased nutrient use efficiency [53]. This is particularly the case for N, as manure has a high N content (12 g kg^−1^ in this study). Cordovil et al. [54] suggested that the nitrogen in organic materials can mineralize to inorganic forms and satisfy the growth demands of plants after amended to soils. Seufert et al. [52] further demonstrated that crops responded more positively to organic amendments under higher nitrogen soil conditions. In addition, Luo et al. [1] indicated that SOC, MBC and MBN are the most important factors with positive effects on crop yield via a structural equation modeling analysis. Compared with CK, soil MBC in this study was significantly increased by 17.66% under the manure treatment. The increase of soil microbial biomass and microbial activity accelerate the acquisition of soil nutrients such as nitrogen and phosphorus by plants [55].

The wheat yield in this study was significantly increased by 7.25% under the biochar treatment compared with CK. Increased crop productivity with biochar soil amendment has been demonstrated in both acidic and alkaline soils [35]. This increase may result from the improved soil quality, such as the higher SOC and available P and K contents, and the larger soil aggregation treated with biochar amendment [5, 56]. Multiple regression analysis showed that SOC and MWD were significantly correlated with wheat yield (Fig. 5). Biochar can maintain a stable presence in soil, and the functional groups on the surface of biochar can combine with mineral ions or Fe and Mn oxides on the surfaces of soil particles and persistently promote soil aggregation [57], which provides a better soil structure for crop growth. The large surface area and porosity of biochar can regulate the supply of soil nutrients by absorption and desorption processes. Soil NH_4_^+^ and NO_3_^−^ contents were reported to decrease by 11% and 10%, respectively, after biochar soil amendment [58], which can reduce soil nutrient loss and increase nutrient use efficiency. Meanwhile, soil P and K availability can be activated by biochar [16], increasing the uptake by crops, which provides a better chemical environment for crop growth. Many studies have reported that biochar soil amendment can increase soil microbial biomass and activity and increase microbial diversity [42]. Improvements in soil microbial and nematode community structure play an important role in soil nutrient cycling and reflect the soil fertility conditions, such as soil nutrient content, soil moisture and soil structure [15, 30], thereby providing a better biological environment for crop growth. This was further proved in the current that wheat yield was significantly correlated to the SOC content and MWD, and a significant indirect correlated with nematode ecology index such as λ and ∑MI.

## Conclusions

Biochar and manure amendments improved the fertility of yellow cinnamon soil after a 5-year treatment. Soil structure was improved as MWD was increased, and soil water and nutrient supply was increased as water content and nutrient contents (such as available P and SOC) were increased compared to CK. The improvement of the soil environment led to a better soil ecosystem, as soil microbial biomass and activity also increased, and an increase in soil nematode abundance and improvement of soil nematode community structure. Biochar and manure amendment enhanced crop productivity, wheat yield was increased by more than 7.25–12.76% compared to CK. The increase in crop productivity was significantly correlated with SOC, MWD, and *Mesorhabditis* sp. and *Pratylenchus* sp. abundance, and the nematode ecological indices ∑MI and H′ served as good indicators of crop productivity in yellow cinnamon soil.

## Methods

### Experimental site

The field experimental site was located in the town of Zhaohe, Nanyang Municipality, Henan Province, China (33°08′ N, 112°58′ E). This area has a subtropical monsoon climate, with average annual precipitation of 704–1173 mm. The soil, classified as yellow cinnamon soil (Xanthic Ali-Udic Cambosols), was developed from Q_3_ loess parent material [59]. Long-term winter wheat and summer maize rotation at the experimental site was arranged before the experiment. The basic properties of the topsoil (0–0.20 m) were pH (H_2_O) of 5.92, soil organic carbon (SOC) of 13.24 g kg^−1^, available N of 191.02 mg kg^−1^, available P of 46.59 mg kg^−1^, available K of 99.00 mg kg^−1^, and soil bulk density of 1.50 g cm^−3^. During the experiment period, winter wheat was sown in early October and harvested in early June, and summer maize was sown in early June and harvested in mid-September.

## Materials

Peanut shell biochar was collected in this field experiment, and the biochar was provided by the Sanli New Energy Company, Henan Province, China. The processes of the biochar production were described in Pan et al. [60]. In brief, the biochar was produced in a vertical kiln at a final pyrolysis temperature of about 500 °C. About 30% of the feedstock biomass was converted to biochar. Other products included about 250 kg of bio-liquid (wood vinegar and pyrolysis oil) and 800 m^3^ of syngas per ton of feedstock. Before applied to soil, the biochar was ground to pass through a 2 mm sieve and homogenized. The properties of the biochar were characterized following the protocol described by Lu [61]. The basic biochar properties were total carbon content of 647 g kg^−1^, total nitrogen content of 15.22 g kg^−1^, and pH (H_2_O) of 9.16, specific surface area of 12.13 m^2^ g^−1^, Ash content of 2.41% and CEC of 148 cmol kg^−1^. The chicken manure used in the experiment was commercial organic manure, which was provided by Xuzhou Hebao Fertilizer Company Limited, the basic properties were total carbon content of 283 g kg^−1^, total nitrogen content of 12.00 g kg^−1^, and pH (H_2_O) of 8.16, total phosphorus content of 19.05 g kg^−1^, total potassium content of 17.83 g kg^−1^, total Cu of 55.6 mg kg^−1^, total Cd of 0.8 mg kg^−1^, total Pb of 21.5 mg kg^−1^ and EC of 5737μS cm^−1^.

### Field experiment

The experiment was begun conducted in 2012. Four treatments were designed in this experiment: (1) CK: conducted as the local conventional fertilization, which applied as mineral fertilizers, (2) Biochar: biochar was applied ar 4.5 t ha^−1^, and applied the same amount of mineral fertilizers to the CK, (3) Manure: manure was applied at a level of 9 t ha^−1^, which replaced 60% of the seasonal mineral N fertilizer of conventional fertilization, and mineral P and K fertilizer was the same with CK, and (4) BM: chicken manure was applied at a level of 9 t ha^−1^, and biochar was applied at 4.5 t ha^−1^, which manure replaced 60% of the mineral N fertilizer of conventional fertilization, and mineral P and K fertilizer was the same with CK. Mineral fertilizers (N, P, and K) were applied as urea, calcium superphosphate, and potassium chloride, respectively. The conventional fertilization was applied at rate of 180 kg N ha^−1^, 90 kg P_2_O_5_ ha^−1^, and 75 kg K_2_O ha^−1^ in wheat season, and at rate of 210 kg N ha^−1^, 75 kg P_2_O_5_ ha^−1^, and 90 kg K_2_O ha^−1^ in maize season. The experiment was conducted in a randomized block design in triplicate. Each plot had an area of 40 m^2^ (5 m × 8 m). The individual plots were separated by a ridge that was 0.5 m wide, and a 135 cm wide protection row was used around the plots. The cropping system during the experiment was wheat–maize rotation, and the varieties of wheat and maize were Zhengmai 9023 and Nonghua 101, which were provided by Hefei Fengle Seed Industry Company Limited and Beijing Jinsenonghua Seed Technology Company Limited, respectively, and the varieties was persisting the same over the experiment period.

The manure and biochar were spread on the surface of soil and then incorporated into the soil by plowing to a depth of 0.2 m before sowing the wheat each year. No biochar or manure was amended during the maize season. Half of the N fertilizer and the total of the P and K fertilizer were applied as base fertilizer. The remaining 50% of the N fertilizer was applied as a dressing at the jointing stage of the wheat season. The fertilization treatment was consistent over the 5 years of the experiment, and the dose of mineral fertilizer, biochar and manure was used for each treatment every year. Other management practices, such as pesticide application and irrigation, were consistent with local farm management.

### Soil sampling and analysis

Soil samples were collected 5 years after the experiment conducted on May 30, 2017, and after wheat harvested. Composite samples of topsoil at 0-0.2 m depth were collected using an Eijkelkamp soil core sampler with an inner diameter of 3 cm, and five randomly chosen soil cores was obtained in each plot. The samples were placed in resealable plastic bags and shipped to the laboratory for further analysis. A portion of each fresh sample was kept at 4 °C for nematode and microbial biomass analysis, and another was air-dried for chemical properties analysis.

Soil properties analysis followed the protocol described by Lu [61]. Soil moisture was measured gravimetrically by drying the samples at 105 °C. Available N was measured using alkaline hydrolysis diffusion method, of which 2.00 g air dried soil was mixed with 10.0 mL 1 mol L^−1^ NaOH solution and incubation at 40 °C for 24 h, then titration with 0.01 mol L^−1^ H_2_SO_4_ solution. Available P was determined using the molybdenum blue method, of which 2.5 g air dried soil was extracted with 50 mL 0.5 mol L^−1^ sodium bicarbonate solution and then measured using a colorimetric method. Exchangeable K was extracted with 1.0 mol L^−1^ ammonium acetate solution (pH 7.0) and determined with a flame photometer (FP6410, Company of Shanghai Jingke, China). Soil organic carbon content was measured using the potassium bichromate titrimetric method. Soil pH was determined using a glass electrode (DZS-707; Zhejiang Nade Scientific Instrument Co., Ltd., Shanghai, China) in a soil:water ratio of 1:2.5 (w:w). Microbial biomass C and N were determined using the chloroform fumigation direct extraction method with correlation factors of K_EC_ = 0.45 and K_EN_ = 0.54 and measured using the potassium bichromate titrimetric method and the semi-Kjeldahl method, respectively. Soil basal respiration was determined using a gas chromatography system (GC-2014, Shimadzu, Kyoto, Japan) based on the linear increase in gas with time [62]. Soil aggregates were dispersed by low-energy sonication, particle size fractions were separated by a combination of wet sieving and centrifuging, as described by Stemmer et al. [63], and the mean weight diameter (MWD) of water stable soil aggregates was calculated.

### Soil nematode extraction and identification

Soil nematodes were extracted using 50.0 g of fresh soil using a modified Baermann method [64]. The nematodes were heat-killed at 60 °C and then preserved in triethanol-amine formaldehyde (TAF) solution. After counting the total nematode abundance, about 100 specimens per sample were randomly selected under a Motic microscope (40× and 400×) with rubber head dropper, and each nematode was identified to the genus level using diagnostic keys [65, 66]. All nematodes were assigned to the following four trophic groups: herbivores, bacterivores, fungivores, and omnivore-predators [67]. Nematode genera were also assigned colonizer–persister (c–p) values of 1–5 corresponding to their positions along the c–p continuum of their life history [68].

### Statistical analysis

Nematode community and soil respiration quotient were characterized by calculating the following specific indices [69, 70]:

1. Shannon's diversity index (H′):
  $$H^{\prime} = - \sum {p_{i} } \text{ln}p_{i} ,$$
  where *p*_*i*_ is the proportion of individuals in the *i*th taxon.
2. Dominance index (λ):
  $$\lambda = \sum {p_{i}^{2} } ,$$
  where *p*_*i*_ is the proportion of individuals in the *i*th taxon.
3. Summed maturity index (∑MI):
  $$\sum {\text{MI}} = {{\left( {\sum {v_{i} f_{i} } } \right)} \mathord{\left/ {\vphantom {{\left( {\sum {v_{i} f_{i} } } \right)} n}} \right. \kern-0pt} n},$$
  where *v*_*i*_ is the *c*–*p* value of both plant-parasitic and free-living nematode taxa, and *fi* is their frequency.
4. Channel index (CI):
  $${\text{CI}} = 100 * {{0.8{\text{Fu}}_{2} } \mathord{\left/ {\vphantom {{0.8{\text{Fu}}_{2} } {\left( {3.2{\text{Ba}}_{1} + 0.8{\text{Fu}}_{2} } \right)}}} \right. \kern-0pt} {\left( {3.2{\text{Ba}}_{1} + 0.8{\text{Fu}}_{2} } \right)}},$$
  where *Ba* and *Fu* are the numbers of bacterivores and fungivores in the total soil nematode population.
5. Respiration quotient (*q*CO_2_), the ratio between soil basal respiration and microbial biomass carbon:
  $$q{\text{CO}}_{2} = {{SR} \mathord{\left/ {\vphantom {{SR} {MBC}}} \right. \kern-0pt} {MBC}},$$
  where SR is soil respiration in g CO_2_-C g^−1^ soil and MBC is soil microbial biomass C.

All data were presented as mean ± standard deviation. An analysis of variance (ANOVA) that considered the two factors of biochar and manure was conducted. The least significant difference (LSD) was used to compare means at the level of 0.05. Statistical analyses of the two-way ANOVA and Pearson correlation coefficients were performed using SPSS 20 software. The relationship between soil nematode assemblages and soil environmental factors was evaluated using redundancy analysis (RDA), which was performed with R software version 3.0.1.

## Data Availability

All data generated or analysed during this study are included in this published article.

## Abbreviations

∑MI: Summed maturity index
AN: Available nitrogen
AP: Available phosphorus
Ba: Bacterivores
BM: Biochar and manure amendment
CI: Channel index
EK: Exchangeable potassium
Fu: Fungivores
H′: Shannon's diversity index
He: Herbivore
MBC: Microbial biomass carbon
MWD: Soil mean weight diameter
Op: Omnivore-predators
SOC: Soil organic carbon
SM: Soil moisture
SR: Soil respiration
Tn: The total nematode abundance
λ: Dominance index
